# Paid caregivers' experiences of falls prevention and care in China's senior care facilities: A phenomenological study

**DOI:** 10.3389/fpubh.2023.973827

**Published:** 2023-02-16

**Authors:** Yuan Luo, Haiye Ran, Yuqian Deng, Huan Li, Mengxi Zhang, Liping Zhao

**Affiliations:** ^1^Xiang Ya Nursing School, Central South University, Changsha, Hunan, China; ^2^Xiang Ya Second Hospital of Central South University, Changsha, Hunan, China

**Keywords:** qualitative analysis, fall prevention, paid caregivers' experiences, senior care facility, phenomenological study

## Abstract

**Background:**

In China, as population aging accelerates, senior care facilities have gradually become a mainstream option. According to the World Health Organization (WHO), the annual rate of falls has increased from 30 to 50% in senior care facilities. A study found that older adults who live in senior care facilities are three times more likely to fall than those who live in the community. The quality of care is highly related to the occurrence of falls. Therefore, exploring paid caregivers' experiences is very important to prevent falls in senior care facilities.

**Objective:**

The aim of this study was to explore paid caregivers' experiences of fall prevention and care in China's senior care facilities. Furthermore, we discussed the situation and provided suggestions.

**Design:**

This is a phenomenological study using face-to-face, in-depth, semi-structured interviews.

**Setting:**

The study was conducted at *four* senior care facilities in Changsha, Hunan, China.

**Participants:**

Fourteen paid caregivers in four senior care facilities, including nursing assistants and senior nurses, participated in this study.

**Methods:**

A purposive sample method was used to select 14 nursing assistants and senior nurses from four different senior care facilities in Changsha from March to April 2022. Every participant individually completed a face-to-face, in-depth, semi-structured interview. Based on the phenomenological research methodology, the thematic analysis method and the Colaizzi analysis method were used for data analysis and theme extraction.

**Results:**

Based on interview data, a total of seven themes were distilled: (1) paid caregivers' professional requirements; (2) paid caregivers' attitude toward falls; (3) paid caregivers' fall training and education; (4) paid caregivers' knowledge about falls; (5) paid caregivers' fall risk assessment; (6) paid caregivers' fall prevention; and (7) paid caregivers' fall treatment.

**Conclusion:**

In China's senior care facilities, paid caregivers to need to be responsible and pay appropriate attention to older adults. First, senior nurses and nursing assistants need to enhance communication and cooperation. Second, they must learn about deficiencies in fall risk assessment and try their best to improve their capability. Third, they must adopt appropriate education methods to improve fall prevention capability. Finally, the protection of privacy should be taken seriously.

## 1. Introduction

In the Global Burden of Diseases, Injuries, and Risk Factors Study 2017, falls were identified as the second cause of death in older adults because of accidental or unintentional injuries worldwide, following road traffic injury (1). In addition, falls were the most common cause of unintentional injuries among older adults (1, 2), including non-fatal injuries such as abrasions or contusions, and fatal injuries such as fractures or head injuries. Although falls could occur in all age groups, age still remains the most critical risk factor for fall-related deaths (3). The majority of fall-related deaths occurred in people over 65 years, especially among people over 70 years (4). It was common that older adults were “silent fallers” who did not report falls and failed to ask for medical care unless injured (5). The risk of recurrent falls increased when falls were not observed and addressed. Falls, particularly in developing countries, were a major unresolved public health problem, which, to some extent, causes health and medical care costs (6).

Senior care facilities are important institutions that provide life care, health and medical care, and psychological care for older adults. In China, as aging accelerates, senior care facilities have gradually become a mainstream choice. According to the World Health Organization (WHO), the annual rate of falls has increased from 30 to 50% in senior care facilities. A study found that older adults who live in senior care facilities are three times more likely to fall than those who live in the community (7). Among the elderly, more than half had at least fallen once in the past year, and nearly 30% had suffered from an injury as a result (8, 9).

In China's senior care facilities, the care of older adults is mostly provided by nursing assistants and senior nurses. The quality of care is highly related to the occurrence of falls. A study illustrated that appropriate concern showed a positive effect on fall prevention. The concern level could be related to the characteristics of nursing assistants and senior nurses and the atmosphere of different facilities (10, 11). For fall prevention, almost all nursing assistants used physical restraint frequently due to a lack of clear prevention strategies. The related research showed that frequent use of physical restraints can increase fall risk and even cause death (12–14). Therefore, exploring paid caregivers' experiences is very important to prevent falls in senior care facilities.

In China, paid caregivers were consistently ignored by researchers when it came to fall prevention in senior care facilities. Based on the phenomenological study method, we would explore paid caregivers' experiences of fall prevention and care in senior care facilities. By summarizing similar viewpoints, we will provide suggestions to guide clinical care better. The findings could provide references about fall prevention and care for senior care facilities in the future.

## 2. Methods

### 2.1. Participants

According to the maximum differentiation principle (15, 16), a purposive sampling method was used to select nursing assistants and senior nurses from four different senior care facilities in Changsha from March to April of 2022. All four senior care facilities were integrated eldercare services with medical care. Two public institutions were located in urban and rural areas, and the others were private institutions including one specializing in rehabilitation and one in cognitive disorders. Inclusion criteria are as follows: (1) working as a paid caregiver in a senior care facility, (2) at least 2 years of care experience in a senior care facility, and (3) informed consent and willingness to participate in this study. Exclusion criteria are as follows: (1) not working for more than 1 month and (2) unfinished interviews. The sample size was based on information saturation, which meant the content of the interviews was repeated and data analysis did not produce any new themes.

### 2.2. Study design

#### 2.2.1. Study instruments

According to the study's purpose, a semi-structured interview outline was developed in discussion with researchers and two geriatric nursing specialists, based on the extensive literature analysis. Pre-interviews were conducted with two interviewees who met the criteria, and the expression of the questions was adjusted based on the results of the pre-interviews. Finally, the interview outline was developed (Table 1). Based on the phenomenological research methodology, first, we asked about the current situation. Then we attempted to explore experiences linked to the environment. Based on the interviewees' responses, appropriate follow-up questions could be asked. Certainly, we also selected the appropriate time to investigate the environment and management of each senior care facility.

**Table 1:** Interview outline.

**Number**	**Interview outline**
1	How old are you? What is your current career? How many years in the career? What was your previous job? What is your highest education?
2	Which factors do you think increase the risk of falls among older adults in senior care facilities?
3	Which is fall risk assessment tool currently used in your facility? What do you think about the effectiveness of this tool? What are the remaining problems?
4	Do you currently make regular fall assessments for older adults in your facility? What are the assessment projects? In order to prevent falls in older adults, what measures can be taken?
5	Is fall prevention education available at your facility? And what have you learned about fall prevention?
6	Can you provide older adults with fall prevention education? Can you tell me what educational content is involved?
7	In your facility, has a fall management system been implemented? What regulations are involved?
8	Please recall the process of recent fall of the older adult (including fall occurrence, handling, reflection and so on).

#### 2.2.2. Data collection

After obtaining the cooperating facility's consent and learning about its characteristics, according to inclusion and exclusion criteria, we would select interviewees (nursing assistants and senior nurses). Based on the phenomenological research methodology, every interviewee completed a face-to-face, in-depth, semi-structured interview. When we did not make offline interviews due to limited conditions, telephone or web video interviews were implemented. First, we introduced the study to interviewees. After obtaining consent, the interviewer and interviewee all determined the time. The environment of the interview was quiet, such as the meeting room. Second, the interviewees were required to sign an informed consent form before the interview. Every interview lasted about 15–20 min. The interview contents were audio-recorded. Finally, the interviewers listened carefully and observed the expressions and attitudinal changes of the interviewees during the interview. Interviewers made appropriate expressions and kept a non-judgmental attitude. If there were any unclear points, we needed further follow-up questions. The study followed the principles of voluntariness, confidentiality, and respect. Interview recordings were numbered and anonymous.

#### 2.2.3. Data analysis

Interview recordings were transcribed to the text within 24–48 h after the interviews were completed. We checked the interview notes and returned them to the interviewee for clarifications and verifications of unclear content to ensure accuracy. NVivo11.0 plus qualitative analysis software was used to analyze data. We chose the thematic analysis method (17) based on the Colaizzi analysis (18): (1) familiarizing Data; (2) generating initial codes; (3) formulating meanings; (4) clustering themes; (5) developing an exhaustive description; (6) producing the report/manuscript; and (7) verifying themes. Two researchers collected and analyzed data. Data were summarized, encoded, generalized, and extracted to classify different themes. When in a dispute, other researchers should make a discussion until the conclusion was approved. According to phenomenological research, we would summarize different themes and analyze the current situation under each theme. Finally, we would discuss viewpoints in the conclusion.

#### 2.2.4. Ethics

This study was approved by the Xiangya Nursing School of Central South University (Ethical number: E202206). During the study, interviewers followed the informed consent principles and respected the privacy and rights of interviewees. At the beginning of the individual interview, all interviewees provided written and verbal informed consent. We would interpret the study purpose, interview questions, transcription procedures, data use, and anonymization. Interviewees could withdraw from the study at any time.

#### 2.2.5. Credibility

Before an interview, we used the purposive sample method to select interviewees based on the principle of maximum differentiation. The interviewees were all front-line caregivers. They were willing to participate and free to speak in the interviews. During the interview, the interviewers listened carefully and observed the expressions and attitudinal changes of the interviewees. Interviewers should not interrupt a conversation deliberately. If there were any unclear points, we asked further follow-up questions. After the interview, interview recordings were transcribed to text and checked within 24–48 h. Researchers attempted to minimize personal bias in the coding and interpretation of data. Researchers only focused on text for theme extraction.

## 3. Results

### 3.1. Interviewee characteristics

A total of 14 caregivers were interviewed from four senior care facilities. There are 10 nursing assistants and four senior nurses, who are all women. The age range of paid caregivers was 23–56 years. The number of years worked was between 2 and 12 years (Table 2).

**Table 2:** The characteristics of caregivers in a senior care facility.

**Number**	**Age**	**Gender**	**Career**	**Working year**	**Education**	**Working experience**	**Facility**
N1	50	Female	Nursing assistant	9	Primary school	Nursing assistant in hospital	①
N2	52	Female	Nursing assistant	3	Middle school	Home caregiver	①
N3	52	Female	Nursing assistant	4	Primary school	Home caregiver	①
N4	53	Female	Nursing assistant	5	Primary school	Unemployed	①
N5	23	Female	Senior nurse	2	Junior college	Fresh Graduates	①
N6	33	Female	Senior nurse	4	University	Nurse in hospital	②
N7	40	Female	Senior nurse	4	University	Nurse in hospital	②
N8	47	Female	Nursing assistant	3	High school	Waitress	③
N9	56	Female	Nursing assistant	12	Middle school	Unemployed	②
N10	40	Female	Nursing assistant	11	Junior college	Worker	③
N11	37	Female	Senior nurse	12	University	Nurse in hospital	③
N12	52	Female	Nursing assistant	6	Primary school	Waitress	④
N13	55	Female	Nursing assistant	5	Primary school	Home caregiver	④
N14	49	Female	Nursing assistant	3	Middle school	Salesman	④

①,a private senior care facility in cognitive disorder;②,a private senior care facility in rehabilitation;③,a rural public senior care facility;④,a urban public senior care facility.

### 3.2. Content analysis

Through the summary and analysis of the interview data, a total of seven themes were distilled. These themes were described and interpreted.

#### 3.2.1. Paid caregivers' professional status

##### 3.2.1.1. Unclear responsibilities

“*China Professional Skills Standard for Nursing Assistants(2019)”* claimed that the work mainly includes daily care, basic nursing, rehabilitation services, psychological care, care assessment, quality management, and training guidance. Nevertheless, most senior care facilities were unable to meet these requirements, and most nursing assistants only provided daily care. N1: “The main work is to provide daily care for older adults.” N4: “Daily care! This is my duty.” N9: “A major part of my job consists of caring for older adults on a daily care.”

Nursing assistants often did not think that their work was relevant to medical-related work. As a consequence, the work of senior nurses was also focused on medical-related nursing and daily care. N1: “Nurses and doctors will make a Careful assessment and examination for older adults.” N13: “I am mainly concerned about older adults' daily life. Others I do less.” In addition to the heavy workload, in senior care facilities, there were few senior nurses, and their roles were unclear, resulting in unclear responsibilities between senior nurses and nursing assistants. N5: “As a nurse, I always check everything.” N6: “Every older adult is cared by senior nurses and nursing assistants within 24h.”

##### 3.2.1.2. Overlapping work

Caregiver care ability played a relevant role in older adults' fall prevention (19–21). It was difficult to provide appropriate care and services for nursing assistants because they could not receive sufficient information. Therefore, nursing assistants would observe older adults all the time and prevent them from falling. A comparable amount of human resources would also be wasted on senior nurses who observed irregularly to ensure the effectiveness of fall prevention. N5: “As a nurse, basically I have to go to every floor...... Usually, we need to check whether pants are too long, whether shoes are well worn, whether the floor is tidy, and whether the fall prevention settings are in good condition. Meantime, we should clean up regularly.” N6: “Essentially, every older person is cared for by a nursing assistant who is a 24-hour caregiver, along with doctors and nurses.”

#### 3.2.2. Paid caregivers' attitude toward falls

##### 3.2.2.1. High level of attention

Falls are very important security incidents in senior care facilities. In daily care, they should pay high attention to the activities and movements of older adults. As part of their daily care, they were fully aware of the physical status of older adults, and they were able to integrate fall prevention throughout their daily care and assisted them with activities of daily living. N4: “I very care about falling……I pay attention to any activities of older adults all the time.” N11: “Paid caregivers must pay more attention to his condition and be aware of any changes. It is our duty to make sure older adults know they are taken care of.”

##### 3.2.2.2. Responsibility

Paid caregivers believed the responsibility was highly related to the falls of older adults. Paid caregivers with a strong sense of responsibility would be more aware of their responsibilities in daily care and pay more attention to their status. N8: “We need to be clear about the duty. With a strong sense of responsibility, paid caregivers are more attentive to the daily routine of older adults in their daily care.” N12: “The most important thing is the sense of responsibility! If you couldn't have it, you wouldn't take good care of older adults.”

##### 3.2.2.3. Keep their eyes open at all times

Fall occurrence is often instantaneous. Paid caregivers need to keep their eyes open at all times. N9: “Keep my eyes on him at all times!” While neglect occurs, older people are at a high risk of falls. N11: “Sometimes a careless caregiver always thinks that older adults will not fall. For example, she may leave when older adult is going to the toilet, and then there are risks in this case.”

During this study, all paid caregivers paid attention to the situation of each older adult in daily life, especially those who were at high risk of falling, such as those with impaired cognitive ability and balance deficits. Paid caregivers will focus more on them all the time. N2: “It is mainly to be deeply concerned for him! If he is at high risk of falls, when he wants to do something, we all need take notice every time.” N5: “If the dementia level is more severe, you should pay more attention to him. For example, when he is moving around, then you should observe if he has a walking stick, and notice if he walks steadily. When he is not in a good condition, you can help him to rest.”

#### 3.2.3. Paid caregivers' fall training and education

##### 3.2.3.1. Insufficient training for caregivers

Although paid caregivers lacked fall knowledge, sufficient training had not been provided. The training forms were mostly lectures, which were held in the morning meeting irregularly. Paid caregivers did not take fall-related knowledge seriously. N6: “We didn't be systematic learning regularly.” N8: “Training mainly requires us to observe them frequently……anyway, there is nothing special.” N9: “I barely take part in training. I can't learn anything from it.”

##### 3.2.3.2. Lack of education for older adults

If older adults could acquire fall-related knowledge, they could notice and prevent falls more effectively. However, paid caregivers could not provide enough fall education for them. The form was mainly a poster that they need to read by themselves, and paid caregivers could remind them irregularly. N9: “It's main a poster.” N6: “We merely particularly organize the training for them. We often remind them to be careful.” N11: “Paid caregivers occasionally remind older adults to be careful.” Some older adults in senior care facilities have different degrees of dementia. Reminding or educating cannot guarantee prevention effects. N2: “There is not much education for older adults because they have decreased cognitive abilities and don't understand these.” N14: “Many older adults suffer from dementia, which makes it difficult to communicate with them. It is difficult for paid caregivers to prevent falls by reminding only.”

##### 3.2.3.3. Less effective oral education

In terms of fall prevention, there was a greater reliance on paid caregivers. Because of their limited capacity, the prevention method was mainly to remind older adults through oral education. N10: “We are always asked for more attention and more reminders for them.” However, some older adults with a high level of self-confidence ignore such reminders, especially those who walk unsteadily. N11: “You just tell him once or twice. He may ignore it. So you have to keep reminding him constantly.” N12: “Especially older adults who walk unsteadily could ignore you. Your reminder didn't work. When you talk with him just now, he may not leave. Once you leave, he will go somewhere.”

#### 3.2.4. Paid caregivers' knowledge about falls

##### 3.2.4.1. Knowledge further enhanced

During the interviews with each paid caregiver, it was concluded that paid caregivers were mostly not confident in their knowledge about falls through their answers, actions, and expressions. N4: “I haven't learned about relevant knowledge (Whisper)……Observe the look, right? Observe his condition, right? If he could move, observe mobility, right? (Look at the interviewer with a lower voice.)”. N8: “You can ask a little. (Embarrassed smile)”. N14: “We are not well educated.”

Paid caregivers were gradually more aware that it was easy for older adults to fall who suffer from multiple chronic illnesses and took multiple medications, but they did not know the relevant details. N3: “It might have an effect on falls about medication.” N7: “There are some older adults who take long-term medication, and part medication effects might cause falls.”

Some paid caregivers thought that some falls were unpredictable, so they usually tended to take measures to limit the high-risk activities for older adults. N9: “Older adults are mainly in the limited area for activities.” N13: “If it is easy to fall for an older adult, we will restrain him.”

##### 3.2.4.2. Rich experience

Although the paid caregivers' knowledge of fall risk and prevention was poor, their rich experience led them to better results in daily care. Therefore, the caregiving experience played an important role in preventing falls among older adults in senior care facilities. N8: “I just rely on my own experience.” N12: “We have lower education. I only have more observations to prevent falls. For so many years, older adults who I take care of barely fall!”

Paid caregivers did not have a knowledge system of fall risk and fall prevention. In daily care, caregivers were often influenced by previous experience. N3: “Sometimes when old adults get up in the morning, and they feel dizzied and unstable walking, it is easy to fall.” N5: “There is water on the ground. Older adults could slip and fall with a greater risk. Then obstacles are the same.” N6: “The older he gets, the higher his fall risk is.”

Paid caregivers took the older adults' living environment seriously. They thought the environment was closely relevant to the occurrence of falls. They would try their best to ensure environmental safety. N1: “Toilets and bathrooms are equipped with anti-slip mats and handrails.” N3: “The bed should be adjusted very low before going to bed (Be convenience for older adults.).”

Paid caregivers found that psychological and social conditions correlated with their falls. N2: “Between two older adults, they conflict with others, sometimes pushing each other, and when we are not in time, he could fall.” N6: “There are also psychological factors, such as irritable temper.” N12: “When he is upset, he will also walk around, and he is also prone to fall.”

#### 3.2.5. Paid caregivers' fall risk assessment

##### 3.2.5.1. No uniform standards

There were no uniform standards for fall risk assessment and no uniform requirement for assessment frequency in each senior care facility. N5: “Generally Morse Fall Scale (MFS) is evaluated once every two weeks, and other related tools are reevaluated once every two months.” N11: “In our facility, we use the Morse Fall Scale (MFS). Generally, each older adult is evaluated at admission, every 3 months, and again if there is a change.” And also there is a lack of fall risk assessment tools in senior care facilities. N9: “We don't have any fall assessment tools. We just observe by ourselves.”

In senior care facilities, the senior nurses made fall risk assessments, and the nursing assistants were not required to do it. N4: “That's a nurse's work, and I rarely do that……It's hard for me to understand those assessments.” N10: “It's nurses' work, not ours.” In daily care, nursing assistants evaluated fall risks mainly through care experience and did not care about fall assessment results. N2: “I just don't remember those assessment details. I mainly observed them.” N12: “I don't know clearly about assessment results generally. I care for older adults by experience. For example, when older adults go to the toilet, we must company with them. We cannot leave until he finishes and helps them back.”

##### 3.2.5.2. Inadequate assessment

Many older adults required long-term multiple medications (22). It was found that paid caregivers would make notes about times, doses, and types of medications. They would remind older adults to take their medications on time. N2: “We have collected some information on times and types of medications, and we will check the medications by this information.” Although paid caregivers understood the relationship between medication and falls, there was an apparent lack of medication factors for assessment and prevention (23). N3: “Sometimes older adults are dizziness after medication. It could be observed.” N5: “There is also a medication factor……I only know taking antihypertensive drugs makes older adults dizzy.”

In addition, the psychological assessment was mostly evaluated based on paid caregivers' experience. N7: “Psychological factors, such as the awareness of fall or fear of fall, are difficult to evaluate……overconfident older adults often think that you don't need to worry about them.” There was a lack of professional psychological assessment tools. N6: “There is no specific tool to assess psychological factor. It is basically based on our own judgment. (Embarrassed smile)”. In addition, there was a lack of a comprehensive approach to caring for older adults with emotional problems, and most paid caregivers just took to them. N4: “An older adult always breaks things every night and I will communicate so little every day that he feels quiet and comfortable.”

Now, it was based more on paid caregivers' observations about the assessment of balance and gait for older adults in senior care facilities, which was not standard and precise enough. There was a lack of professional staff and assessment tools. N7: “I think it (the nurse's assessment) may not be too precise in the assessment of gait and balance. We just observe the walk situation.” N11: “The assessment of gait relies mainly on our own observations simply……without a further professional assessment.”

Paid caregivers also think that it was difficult to prevent falls in some special conditions. N8: “I can't control falls, and I can't bind older adults to me!” Observations were still essential to learn about the life habits of older adults, especially toileting and bathing, which were common causes of falls in senior care facilities. N3: “But some things are difficult to evaluate. That is to say, sometimes he goes to the toilet on his own. It is easy to slip and fall.”

#### 3.2.6. Paid caregivers' fall prevention

##### 3.2.6.1. Daily care

At present, life care was the main work in senior care facilities, which was mainly provided by nursing assistants. Paid caregivers often combined fall prevention with their daily care. N1: “Fall prevention is often about the three-time points. From 5 to 7 a.m. in the morning, older adults are easy to fall. From 12 to 14 p.m., older adults will take a bath. It is easy to fall. From 17 to 19 p.m. in the evening, is the time when older adults go to bed. They are also prone to fall.” N9: “My main work is to take care of older adults and how to prevent falls in daily care.”

In daily care, paid caregivers believed that the appropriateness of dress is related to falls. They often observed whether the pants were too long and whether the shoes have the right fit. N1: “The first thing is suitable clothes, where the pants can't be too long, and non-slip shoes are the right size.” They remind families to prepare non-slip shoes for older adults. N3: “We will explain to families to buy non-slip shoes and check the suitability of shoes.”

##### 3.2.6.2. Older adults' feeling

Many older adults who just moved into senior care facilities are unaccustomed and uncomfortable at first (24), which was one of the causes of falls, and even some might be violent and cause others to fall. N2: “Between two older adults, they conflict with others, sometimes pushing each other, and when we are not in time, he could fall.” In daily care, paid caregivers often observed the emotional changes of older adults and noticed more. N3: “Whenever another older adult is near his room, he gets angry...... Sometimes he is in a bad mood, so we keep an eye on him.”

#### 3.2.7. Paid caregivers' fall treatment

##### 3.2.7.1. Comprehensive post-fall treatment

Post-fall treatment was taken very seriously in senior care facilities. Paid caregivers often learned how to address falls. First of all, paid caregivers were required to check the condition. When the condition was unknown, they forbade picking up older adults. Second, they need to call nurses and doctors to check again. If an older adult was not serious, they should take the older adult to rest in bed and pay attention to the changes within 24 h. If not, older adults should seek medical treatment in time. N1: “When an older adult falls, we are the first to check him and ask him where the pain is. Immediately we will inform nurses and doctors. They will make a detailed assessment. If the assessment result is good, we will take him to rest. If not, doctors need to observe whether he has a fracture or something and decide whether it is necessary to the hospital.” Finally, they will reflect too carefully on the fall condition to avoid such incidents. N5: “If an older adult falls, we will watch and analyze a fall video and then find the causes of the fall.” N7: “After the fall, our department makes a reflection to analyze some causes and summarize some experiences.”

In senior care facilities, most nursing assistants first found falls. Due to their lack of knowledge, they only accompanied and comforted older adults to prevent them from secondary injuries. N4: “We will comfort older adults and prevent them from secondary injuries.” N14: “After the fall, we will comfort older adults. Let them not be anxious and stressed.”

## 4. Conclusion

### 4.1. Communication and cooperation

There is a lack of caregivers in China's senior care facilities. In addition, the number of nursing assistants is larger than senior nurses. Several studies have shown that care quality was significantly affected by caregiver level, and falls were associated with the number and structure of the caregivers (25–27). An appropriate level of caregivers could decrease the rate of falls.

In senior care facilities, senior nurses make a fall risk assessment but nursing assistants prevent falls in daily care. Due to poor communication, nursing assistants only do their own work, despite the critical information known only by the senior nurses. In addition to the background of human resource shortages, it leads to an increased burden and wasted labor force.

Therefore, managers should make regulations to enhance cooperation. For fall prevention, paid caregivers should communicate with each other to complement work. In the future, we need to explore the number and level of paid caregivers in senior care facilities. Meantime, methods to balance the structure of paid caregivers might be a worthy exploring topic in the future.

### 4.2. Importance of responsibility

In senior care facilities, daily care is very busy and trivial work. Especially for the life quality of older adults, the details often determine success or failure. Fall prevention among older adults is reflected in all aspects of daily care. Therefore, the responsibility sense of paid caregivers is particularly unique.

Falls are common among older adults. Most falls in older adults were preventable and manageable (28). According to content analysis, responsible paid caregivers had a strong belief in fall prevention and believed they could protect effectively older adults from falls. But it was found that some paid caregivers wanted to attribute the cause to an accident, and they also thought their work was too busy to keep an eye on older adults all the time. Paid caregivers could unintentionally ascribe accountability for personal actions to others. Especially senior nurses were primarily responsible for medical services and nursing assistants were mainly responsible for daily care. It was, therefore, essential for paid caregivers to escape the cycle of diffused responsibility (29, 30).

Senior-centered services are gradually becoming more important as humanistic literacy improves. The sense of paid caregivers' responsibility is closely related to the level of care quality. If paid caregivers are careless, older adults are at high risk for falls. As a result, responsible paid caregivers will pay attention to the smallest details so that they can protect older adults from falls. The managers should figure out how to strengthen responsibility and enhance belief.

### 4.3. Appropriateness of attention on falls

In this study, we found that paid caregivers need to observe constantly for fall prevention. They had to check fall-related details comprehensively such as length of pants, suitability of shoes, conditions of daily activities, and so on, but non-systematic observation could easily lead to neglect, and a substantial workload leads more or less to ignoring affairs that cause falls for older adults. After an older adult fell, paid caregivers need to pay more attention to him for at least 24 h, which increased the care burden and ignore others to some extent. This, therefore, led to a vicious circle for paid caregivers. Finally, they had to choose the use of physical restraints. A few pieces of research showed that frequent use of physical restraints could increase fall risk and even cause death (12–14). Several studies found that even though paid caregivers acknowledged the potentially serious consequences of the use of physical restraint, they still chose to use restraint to ensure older adults' safety, complete other work, prevent harm to self, and reduce the risk of liability (31–33).

In short, appropriate attention would ask paid caregivers to know what details of older adults need to be noticed. They do not worry too much about the frequent use of physical restraints. Furthermore, we need a uniform fall prevention regulation to guide paid caregivers. The regulation could make paid caregivers prevent falls clearly and systematically.

### 4.4. Enhanced fall education

Sufficient training had not been provided for paid caregivers. Training forms were mainly lectures, which were held mostly in the morning meeting. Because paid caregivers, especially nursing assistants, had not acquired systematic knowledge of geriatric care, they had difficulties in improving care quality through existing training forms. In this study, we found that nursing assistants evaluated fall risks only through care experience and integrated fall prevention with life care. Paid caregivers were often trained on how to address post-fall by situation simulation, which led to good results. It was shown that nursing assistants could learn about fall risk and prevention through situations and experiences. Therefore, it might be worthwhile to explore and apply the situational simulation method further as a teaching method.

In China's senior care facilities, there are no effective fall education methods for older adults commonly. Only through posters and paid caregivers' reminders, it could not be effective. Many older adults even ignore reminders and reject care, and it could bring about combative or physically aggressive behaviors if caregivers persisted in providing the rejected care despite patient refusal (34). Some older adults had different degrees of dementia which was a strong risk factor for falls that the paid caregiver's core competence would be required at a higher level (35).

### 4.5. Improvements in fall risk assessment capability

In China's senior care facilities, fall risk assessment is mostly undertaken by senior nurses, and the number of senior nurses in senior care facilities is small at present. In daily care, most nursing assistants were not required to make a fall risk assessment. They tend to assess fall risk through care experience.

According to an analysis, that phenomenon is caused by the following three main reasons: (1) Nursing assistants merely systematically acquire knowledge of geriatric nursing, and their working experience is barely related to geriatric care; (2) MFS is the most commonly used assessment tool in four senior care facilities. Because it is primarily used in hospitals, it is controversial about the value of assessing fall risk for older adults in senior care facilities (36). It was, however, recognized that the MFS did not screen for accidental and unanticipated physiologic falls because these were unpredictable events (37, 38). Nursing assistants could observe to prevent accidental and unanticipated physiologic falls. There was, therefore, a lack of fall risk assessment tools for nursing assistants. (3) In most cases, there was a lack of communication between senior nurses and nursing assistants. When senior nurses finished the fall risk assessment, they did not explain fall risk factors in detail, and they only informed nursing assistants about whether older adults were at high risk. Moreover, nursing assistants also did not analyze assessment items and results. As a result, nursing assistants have difficulties in targeting fall prevention by uniform standards.

However, managers should be fully aware of the distribution and cooperation among paid caregivers. Especially, regulation needs to be made for the cooperation of fall prevention. In the future, a unique tool for nursing assistants should be developed. When nursing assistants use an appropriate assessment tool, they find risk factors and make measures precisely.

### 4.6. Protection of privacy

All the time, the protection of privacy is ignored in daily care. Because paid caregivers are afraid of fall accidents, they keep their eyes on older adults to prevent falls including toileting and bathing. When an older adult goes to the toilet or bath, paid caregivers stand at the door and even accompany them inward. A spy to observe could make older adults uncomfortable within 24 h. In some situations, they even restrict activities and interactions.

A study shows that Chinese older adults valued psychological and social privacy more, and less emphasis was placed on physical and informational privacy (39). In the study, older adults experienced a lack of privacy in physical exposure and personal care (40). Quality of care is not only safety but also humanistic care. Especially, paid caregivers are mostly women. The older men feel uncomfortable and shy in intimate interactions. Paid caregivers cannot only finish work and ignore other needs but also need to protect privacy.

## 5. Significance and limitations

This study aimed to explore the common experience of different senior care facilities. The purposive sample method was adapted to select interviewees based on the principle of maximum differentiation. We collected viewpoints from different perspectives. In the study, there were no male caregivers in four facilities. We only explored female caregivers' experiences. But in real life, women are the main caregivers in senior care facilities. The results are still representative. Finally, the facilities are all from Changsha, and in the future, we could compare caregivers' experiences from other cities.

## Data availability statement

The original contributions presented in the study are included in the article/supplementary material, further inquiries can be directed to the corresponding author.

## Ethics statement

Written informed consent was obtained from the individual(s) for the publication of any potentially identifiable images or data included in this article.

## Author contributions

YL, HR, LZ, and MZ: study design and article revision. YL, HR, YD, and HL: qualitative interview and data analysis. YL and HR: article writing. MZ and LZ: article guide. All authors contributed to the article and approved the submitted version.
